# Ultraviolet Grafting of Bismuth Oxide Enhances the Photocatalytic Performance of PVDF Membrane and Improves the Problem of Membrane Fouling

**DOI:** 10.3390/polym16162322

**Published:** 2024-08-16

**Authors:** Chang Liu, Yuxuan Kong, Guojiang Xia, Xiancheng Ren, Jing Zhang

**Affiliations:** 1 College of Polymer Science and Engineering, Sichuan University, Chengdu 610065, China; 2 College of Architecture and Environment, Sichuan University, Chengdu 610065, China

**Keywords:** PVDF membrane, PVDF-g-BA, photocatalytic, anti-fouling performance, self cleaning

## Abstract

Photocatalytic membranes are crucial in addressing membrane fouling issues. However, the grafting amount of the catalyst on the membrane often becomes a key factor in restricting the membrane’s self-cleaning capability. To address the challenge, this manuscript proposes a method for solving membrane fouling, featuring high grafting rates of bismuth oxide (Bi_2_O_3_) and acrylic acid (AA), significant contaminant degradation capability, and reusability. A highly photocatalytic self-cleaning microfiltration membrane made of polyvinylidene fluoride bismuth oxide and acrylic acid (PVDF-g-BA) was prepared by attaching nano Bi_2_O_3_ and acrylic acid onto the polyvinylidene fluoride membrane through adsorption/deposition and UV grafting polymerization. Compared with pure membranes and pure acrylic grafted membranes (PVDF-g-AA), the modified membrane grafted with 0.5% bismuth oxide not only improves the grafting rate and filtration performance, but also has higher self-cleaning ability. Furthermore, the degradation effect of this membrane on the organic dye methyl violet 2B under visible light irradiation is very significant, with a degradation rate reaching 90% and almost complete degradation after 12 h. Finally, after repeated filtration and photocatalysis, the membrane can still significantly degrade contaminants and can be reused.

## 1. Introduction

Recently, water environments are grappling with a significant challenge: dye contamination. This arises from the expansion of textile industries and printing and dyeing factories, which produce wastewater containing non-biodegradable dyes, rendering their treatment exceptionally challenging [1,2]. Methyl violet 2B is among the most frequently utilized dyes, often cited for contaminating rivers and lakes [3,4], leading to significant damage to ecosystems and posing potential risks to human health [5,6,7,8]. Hence, there is an urgent need to develop effective methods for removing methyl violet 2B dye from contaminated water. Various approaches, including physical, chemical, and biological strategies, have been utilized to treat contaminated water through adsorption or degradation processes [9,10,11]. Among these methods, photocatalytic degradation has garnered significant attention due to its high efficiency in degrading contaminants, energy-saving characteristics, and environmentally friendly nature. Particularly noteworthy is the integration of photocatalysts onto the membrane surface, offering a promising avenue for enhancing anti-fouling resistance and facilitating the degradation of organic contaminants in feed solutions through photodegradation.

Advanced anti-fouling membranes provide insights into addressing the current bottlenecks in organic dye wastewater treatment technology and endow membrane materials with new functionalities, such as high permeability, photocatalytic activity, and anti-fouling performance. Bi-based catalysts are commonly used to develop new photocatalytic anti-fouling membranes for the removal of organic wastes like dyes. Among them, Bi_2_O_3_ [12] stands out among many Bi-based semiconductor materials due to its low cost, direct bandgap of 2.5–2.8 eV, and good photostability under acidic conditions. For instance, Li et al. [13] successfully prepared Bi_2_O_3_/ZnS-CA photocatalytic membranes using Bi_2_O_3_/ZnS heterojunctions as photocatalysts through a phase transformation method. The Bi_2_O_3_/ZnS heterojunction structure enhances the separation efficiency of photogenerated charges, resulting in high photocatalytic activity for the degradation of rhodamine B (RhB) under visible light, with an improvement in degradation efficiency of approximately 30%. Additionally, the membrane exhibits excellent permeation flux (649.7 L·m^−2^·h^−1^) and high porosity (69.94%). The Bi_2_O_3_/ZnS-CA membrane also demonstrates good RhB adsorption performance, enhancing the interaction between RhB and the catalyst. Although the above method promotes the photocatalytic degradation of dyes, it does not effectively improve light absorption or significantly enhance anti-fouling capability.

PVDF membranes, as commonly used catalytic membrane substrates, exhibit strong stability but have poor filtration performance and fouling resistance. Therefore, modification is needed to enhance their performance. Advanced photocatalytic membrane technology has provided insights into eliminating the bottlenecks experienced in current membrane technology. It has also endowed membrane materials with new functionalities such as high permeability, fouling resistance, and photocatalytic activity. Multiple strategies have been investigated for their construction, including the self-assembly of nanomaterials, blending photocatalysts, and surface modification [14,15] to create free-standing membranes [16]. Among these methods, blending photocatalysts stands out for its easy preparation, making it a widely adopted economic modification approach [17,18]. For example, Xu et al. [19] successfully prepared hybrid ultrafiltration membranes by blending GO/TiO_2_ with PVDF membrane, exhibiting preferable photocatalytic properties. However, as far as we know, the degradation performance of the GO/TiO_2_ powder catalyst is significantly higher compared to that of the GO/TiO_2_-PVDF membranes. This difference is attributed to the abundance of effective photocatalysts buried within the polymer matrix and the reduction in photocatalytic active sites due to the blending modification. In practice, by modifying the surface of the membrane and loading the photocatalyst onto it, the photocatalytic activity of the membrane can be improved, while the membrane surface also possesses anti-fouling [20,21,22,23] and self-cleaning [24,25,26,27,28] abilities.

The thin liquid-phase ultraviolet (UV) grafting method [29] is a technique for functionalizing the surface of polymer materials. It involves grafting monomers or catalysts onto the surface of polymer substrates in a thin liquid phase under UV irradiation. This method is widely used in materials science and engineering to endow polymer membranes with new functions, such as enhanced hydrophilicity and increased anti-fouling performance. In this paper, AA was used as a hydrophilic monomer, which was dispersed with Bi_2_O_3_ using ultrasonic wave and then grafted upon the surface of PVDF membrane via UV irradiation. The PVDF-g-BA membrane, which integrates membrane separation, photocatalytic degradation, and self-cleaning ability, was successfully prepared.

## 2. Materials and Methods

### 2.1. Materials

Bismuth trioxide (Bi_2_O_3_, particle size = 100 nm) was obtained from Shanghai Naiou Nano Technology Co., Ltd. (Shanghai, China); Polyvinylidene fluoride (PVDF, pore size = 0.45 μm; diameter = 90 mm) membrane for Haining Kezhun Filter Equipment Co., Ltd. (Haining, China); Acrylic acid (AA) from Chengdu Cologne Chemical Products Co., Ltd. (Chengdu, China); Benzophenone (BP) from Tianjin Bodi Chemical Co., Ltd. (Tianjin, China); Dyes, including methyl violet 2B (MV), methylene blue (MB), rhodamine B (RhB), and acid orange 7 (AO7) from Chengdu Kelong Chemical Reagent Factory (Chengdu, China); and Tertiary butyl alcohol (TBA) from Chengdu Kelong Chemical Reagent Factory (Chengdu, China); L-Tryptophan, Chengdu Kelong Chemical Reagent Factory (Chengdu, China).

### 2.2. Preparation of PVDF-g-BA Membrane

By using the UV irradiation grafting technique, a series of modified PVDF membranes were prepared in this experiment. The preparation process is shown in Figure S1. Initially, pure PVDF (M0) membranes with uniform pore size were washed with deionized water, placed in a 70 °C drying oven for 5 min, weighed, and then set aside in a Petri dish. A mixture of AA (20 wt%) and varying concentrations of Bi_2_O_3_ (0.1, 0.25, 0.5, 0.75, and 0.9%) was prepared by adding water to make up to 50 mL in a 100 mL beaker and subjecting it to ultrasonic dispersion in an ultrasonic instrument for 15 min. During this process, a measured quantity of ethylene glycol (molar ratio to the monomer was 1:6.5) and photoinitiator BP (1 wt% of monomer content) were introduced into the ultrasonic dispersed solution with Bi_2_O_3_ and AA. After achieving uniform dispersion, the solution was poured into a Petri dish containing the pure PVDF membranes and soaked for 40 min. The catalyst in the solution was observed to gradually adsorb and deposit onto the membrane surface. Upon removal from the solution, the membrane was transferred to another clean Petri dish, and any air bubbles and excess liquid were eliminated under a roller. Then Petri dish was positioned beneath a UV aging chamber, with a vertical distance of 20 cm from the 400 W UV lamp, and illuminated for 20 min. This process facilitated the grafting of AA and Bi_2_O_3_ upon the surface of polymer membrane. Following UV irradiation, these modified membranes were submerged in distilled water for a day to eliminate impurities and residual reactants on the surface. Finally, the membranes underwent drying in a 70 °C drying oven for 20 min and then were weighed. In the preparation of AA-modified PVDF-g-AA (M1) membrane, the procedure is the same as that of the AA-Bi_2_O_3_-modified PVDF-g-BA (M2–M6) membrane. The only difference is the absence of Bi_2_O_3_ throughout the ultrasonic dispersion when preparing M1. The detailed compositions and nomenclature of modified membranes M0-M6 are depicted in Table S1.

### 2.3. Characterization

ICP-OES was measured with a Agilent 5110 detector from Leiden Scientific Instruments (Suzhou, China); the formula for calculating the rate of each component grafting is displayed in Text S1. XRD signals of samples were recorded by using a Ultima IV X-ray diffractometer (Rigaku, Tokyo, Japan). Water contact angle (WCA) was measured with a DSA100 detector (Kruss, Hamburg, Germany). Energy-dispersive X-ray spectroscopy (EDS) mapping and scanning electron microscopy (SEM) images were measured with a JMS-7500F SEM (JEOL, Tokyo, Japan). FTIR spectrum was obtained with a Nicolet Avatar 370 infrared spectrometer (ThermoFisher, Waltham, MA, USA). UV-vis absorption spectrum was measured with a UV-2000 spectrophotometer from Unico Instruments (Shanghai, China). Brunauer–Emmett–Teller (BET) was measured with a ASAP2460 detector (Micromeritics, Norcross, GA, USA). Zeta potential was detected with a Nicomp Z3000 analyzer from Alpharmaca Inc. (Shanghai, China). The electron paramagnetic resonance (EPR) patterns were obtained with a Bruker model ER200-SRC spectrometer (Bruker, Billerica, MA, USA).

### 2.4. Membrane Anti-Fouling Performance and Mechanism Testing

The PVDF-g-BA membrane anti-fouling performance was measured by adsorption and photocatalytic degradation of MV (concentration = 10 ppm, maximum absorbance wavelength = 584 nm). In a standard photocatalytic measurement, the membrane (20 mg) was cut into four even pieces and added to one of the four 100 mL of the dye contaminants in a beaker. The 40 W visible LED light was used as the light source. Absorbance during the adsorption and degradation processes was recorded every half hour using a UV-vis spectrometer.

At the same time, in order to assess the reuse performance of the modified membranes and characterize their anti-fouling performance, the modified membranes underwent five consecutive photocatalytic degradation cycles. During each cycle, the water flux data were measured and compared with the initial data to obtain the membrane flux recovery rate, defined as Flux/Flux_0_. To further confirm the recyclability of the photocatalytic membrane, the modified membrane M4, which was stirred magnetically in darkness for 30 min to attain adsorption–desorption equilibrium, was subjected to five consecutive cycles of visible light irradiation for the degradation of methyl violet 2B dye. The catalytic degradation time for each cycle was 3 h. After each cycle, the membrane surface was washed with ethanol and deionized water and then dried before continuing with the dye treatment until all five cycles of degradation were completed.

To clarify the self-cleaning mechanism of the modified membranes, reactive oxygen species (ROS) were identified by performing EPR spin trapping experiments and free radical trapping experiments. Multiple radical scavengers were adopted to confirm the self-cleaning mechanism.

## 3. Results and Discussion

### 3.1. Evaluation of Membrane Properties

#### 3.1.1. Characteristics of Modified PVDF Membranes

**Figure 1:**
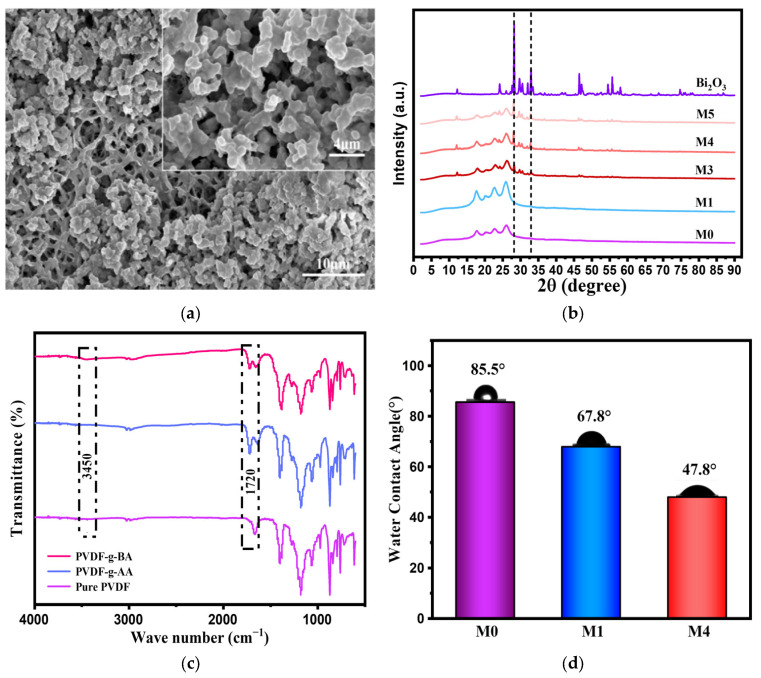
(**a**) SEM images and EDS mapping of M4 before adsorption (the inset shows the image at a higher magnification); (**b**) XRD images of catalyst and membranes with 5 different catalyst contents; (**c**) FTIR images of three types of membranes; (**d**) WCA image of three types of membranes.

Compared with the left SEM image of M0 in Figure S2, the originally smooth and regular fiber surface was covered with a layer of catalyst particles (Figure 1a), making the fiber surface become rough and uneven. This indicates that the catalyst successfully adhered to the membrane surface, altering its physical structure. Moreover, as the amount of catalyst increased, the color of the membrane surface gradually turned a deeper yellow, as shown in Figure S3. From the magnified image in Figure 1a, it can be observed that, due to the increased loading of bismuth oxide catalyst on the membrane, a more uniform and dense layer of bismuth oxide particles has formed. However, as depicted in Figure S3, if the catalyst concentration on the membrane surface is further increased, nanoparticles tend to agglomerate on the membrane surface, leading to severe pore blockage and a significant reduction in the filtration and anti-fouling performance of the modified membrane. Therefore, membranes with either excessively low (M2) or high (M6) catalyst concentrations cannot be used effectively. Figure 1b presents an X-ray diffraction analysis of various membranes. It can be seen that there are obvious diffraction peaks in the dotted frame. The characteristic diffraction peak of the pure PVDF membrane appears between 17° and 26°. Compared with M0 and M1, the diffraction peak gradually increases with the increase in the photocatalyst content around 28° and 33°, and compared with the standard card, it basically accorded with the characteristic diffraction peak of the Bi_2_O_3_ tetragonal crystal system, indicating that the photocatalyst has been successfully loaded on the membrane. Next, Figure 1c is the Fourier infrared (FTIR) spectrum of the membranes. The dotted frame indicates the stretching vibration peak of 1720 cm^−1^ carbonyl group and the stretching vibration broad peak of 3450 cm^−1^ oxygen–hydrogen bond on carboxyl group. In addition, through the EDS images, the elemental distribution on the membrane surface after grafting the catalyst can be analyzed in detail. Figure S4 shows the content and distribution of a total of four elements: C, O, F, and Bi. Among them, the carbon element has the highest content, accounting for nearly half of the four elements. The fluorine element has the lowest content because the PVDF membrane itself has minimal exposed fluorine on its surface. The images indicate that after grafting the catalyst onto the PVDF membrane, a large amount of bismuth element appeared on the membrane surface, and the bismuth element is uniformly and widely distributed.

In summary, the above four sets of data indicate that acrylic acid and Bi_2_O_3_ were successfully grafted onto the membrane surface.

#### 3.1.2. Filtering Performance of Membranes

**Figure 2:**
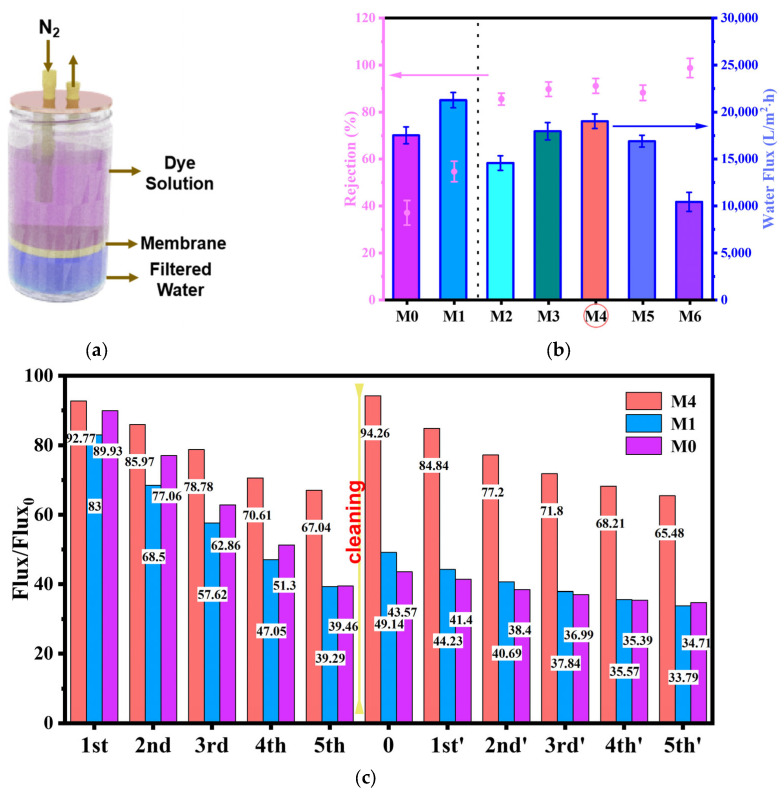
(**a**) The brief diagram of ultra-filtration cup filtration system; (**b**) pure water flux and crystal violet dye rejection ratio of three PVDF membranes with different monomer and catalyst content; (**c**) the ratio of flux to initial flux after five consecutive times filtration of MV with M0, M1, and M4, and the change in the ratio after visible light irradiation.

The filtration performance of a membrane, including flux and rejection ratio, has always been an important criterion for evaluating the quality of a membrane. The ultrafiltration cup filtration system was used to test the pure water flux and the dye rejection ratio under constant pressure through the membranes (Figure 2a). The water flux of the prepared membranes exhibit relatively stable performance under continuous filtration in Figure S5. As shown in Table S2, the modified membranes exhibited rejection capabilities for all four dyes. The dyes, sorted by rejection ratio through the modified membrane M4 from lowest to highest, are AO7, RhB, MB, and MV. The modified membrane loaded with acrylic acid and bismuth oxide showed the greatest improvement in rejection performance for MV dye, with an increase of nearly 57%, indicating good adsorption performance for methyl violet dye. The results, which show the filtration performance of the modified PVDF membrane, has been greatly improved compared with the pure membrane, as shown in Figure 2b. After a series of tests, it is found that the introduction of the photocatalyst and acrylic acid can greatly improve the rejection performance of the membrane, and the rejection ratio of M4 crystal violet dye has increased from 36% of M0 to more than 90%. From the figure, it also can be seen that first, due to the introduction of acrylic acid, the water flux of membrane M1 was improved, increasing from around 18,000 L/m^2^·h of the original membrane M0 to above 20,000 L/m^2^·h. This improvement occurs because acrylic acid, a hydrophilic monomer, increases the hydrophilicity of the originally hydrophobic polyvinylidene fluoride membrane when grafted onto its surface. Secondly, as the amount of catalyst grafted onto the membrane surface increases, the dye rejection ratio of membranes M2–M6 also increases. However, as shown in Figure 1a and Figure S3, an excessive catalyst can lead to severe membrane pore blockage, which is reflected in Figure 2b as a significant reduction in water flux. Compared to M0, the pure water flux of the modified membrane M4 did not change significantly, remaining at a high level of 18,000 L/m^2^·h, but the rejection ratio of MV dye was greatly improved. This is because, even though the loading of Bi_2_O_3_ particles leads to pore blockage, the introduction of polyacrylic acid also enhances hydrophilicity and can restore the flux. To prove this phenomenon, WCA contact angle measurements were performed for membranes M0, M1, and M4. As shown in Figure 1d, it is evident that with the introduction of bismuth oxide and acrylic acid, the water contact angle gradually decreases, indicating the improved hydrophilicity of the membrane. Additionally, membrane M4 has a smaller water contact angle compared to M1, indicating that more hydrophilic acrylic acid monomers were grafted onto its surface, which also supports the grafting rate data presented in this paper. Therefore, M0, M1, and M4 are used for the following analyses and tests. The grafting rates of Bi_2_O_3_ and AA on the membrane surface were calculated by ICP test, and the results are shown in Table 1.

Such high grafting rate confirmed the reason for the excellent filtration performance of the modified membrane.

Subsequently, Figure 2c shows the change in the ratio of the water flux to initial flux after filtering the methyl violet 2B dye solution for five consecutive times and the ratio after visible light irradiation. The results showed that the photocatalytic membrane maintained a relatively stable water flux during the continuous filtration of the dye solution. When continuously filtering methyl violet 2B dye, the water flux of the M4 membrane showed the smallest decrease among the three membranes, whether before or after illumination, and the water flux dropped by about 30%. After continuous filtration, the membrane flux of both M0 and M1 membranes dropped by more than 60%, and the filtration performance was significantly reduced. Both membrane flux recovery rates were less than 10% after illumination. Even after repeated use, the filtration performance of the modified membrane M4 remains in a relatively good state, and continuous filtration has little impact on it. Moreover, after the modified membrane M4 is exposed to visible light, the membrane flux recovery rate reaches 27.22%. The water flux recovery rate is high, indicating that the modified membrane has a certain anti-fouling performance and the ability to be recycled and reused, and has high reusability.

#### 3.1.3. Anti-Fouling Performance

**Figure 3:**
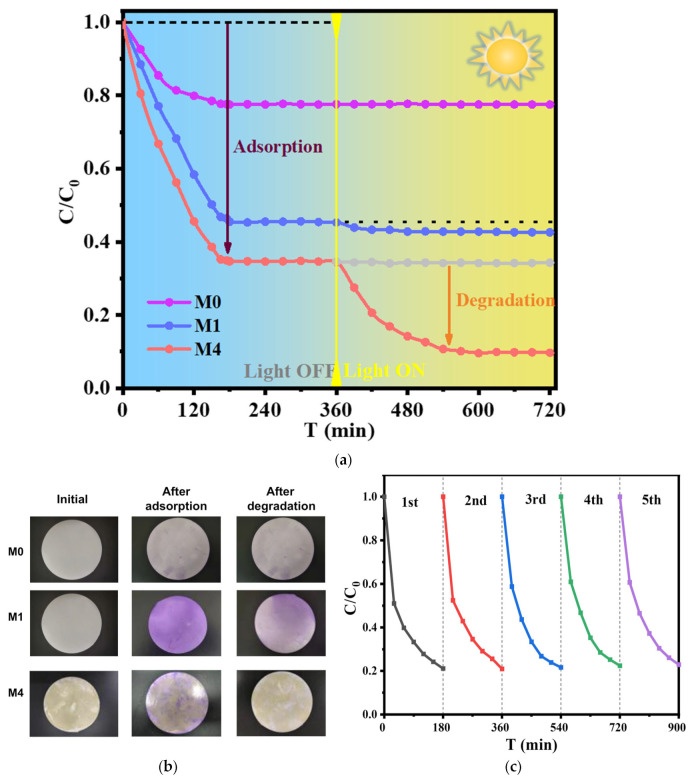
(**a**) Adsorption and degradation of MV by three types of membranes; (**b**) the color changes of M0, M1, and M4 membrane surfaces after adsorption and degradation of methyl violet dye; (**c**) the degradation performance of the M4 membrane after five cycles.

Using crystal violet 2B dye as a model organic contaminant, the photocatalytic performance of the modified PVDF-g-BA membrane is tested. Figure 3a shows the degradation ratio of 10 mg/L crystal violet dye for pure and modified membranes, where C is the concentration of crystal violet after visible light irradiation and C_0_ is the concentration of crystal violet dynamically adsorbed for 1 h in the dark. It can be clearly seen that before visible light irradiation, the modified membrane first removed a portion of the dye by adsorption under dark conditions for 6 h. Subsequently, under the action of the photocatalyst, the modified membrane further degraded the remaining dye in the methyl violet solution and the dye adsorbed on the membrane surface under light conditions. It is evident that the pure membrane M0 only had a 20% adsorption degradation rate for the dye after 6 h and showed almost no further degradation of dye contaminants under visible light. The membrane M1 adsorbed a significant amount of methyl violet, with an adsorption degradation rate of 55%, but showed little change after irradiation, with a degradation rate of less than 5%. In contrast, the modified membrane M4 continued to degrade contaminants under visible light even after reaching adsorption–desorption equilibrium, achieving a final degradation rate of up to 90% for methyl violet dye, exhibiting significant photocatalytic activity.

Figure 3b shows the changes in MV contaminants on the membrane surface after three membranes undergo adsorption and photocatalytic degradation. It was observed that during the treatment of dye solutions, the surface color of membranes M1 and M4 changed significantly, while the color change in membrane M0 was not noticeable due to its smaller adsorption amount. Initially, this change was mainly due to the membrane’s adsorption of the dye. However, under light conditions, the photocatalytic membrane M4, after reaching adsorption–desorption equilibrium, further achieved photocatalytic degradation of the dye, significantly reducing the dye concentration. Additionally, after further illumination, the membrane surface color almost returned to its initial state, demonstrating a certain degree of reusability.

As can be seen in Figure 3c, it is shown that after five cycles, the M4 membrane still has an excellent ability for the catalytic degradation of MV, which demonstrates exceedingly good anti-fouling performance. During this period, we performed SEM analysis on the membrane surface after the first, third, and fifth degradations. As shown in Figure S6, we found that the surface morphology and structure did not change much, and the Bi_2_O_3_ particles were still firmly attached to the membrane surface, showing good stability.

In summary, compared to previous work [30], under LED light irradiation, the modified membrane shows significant adsorption and photocatalytic degradation ability for dyes and demonstrates excellent reusability and high anti-fouling performance in continuous contaminant treatment processes.

### 3.2. Adsorption Mechanism

There are several classical theories for the adsorption mechanism, which include physical and chemical adsorption. On the one hand, physical adsorption is primarily driven by Van der Waals forces and electrostatic attraction. Chemical adsorption, on the other hand, involves the formation of chemical bonds through electron transfer, exchange, or co-ownership between adsorbent molecules and atoms or molecules on the solid surface of the adsorbent. In order to elucidate the adsorption mechanism, numerous theoretical calculations and experiments were carried out.

#### 3.2.1. Capacity of Physical Adsorption

##### The Attraction of Van der Waals

**Figure 4:**
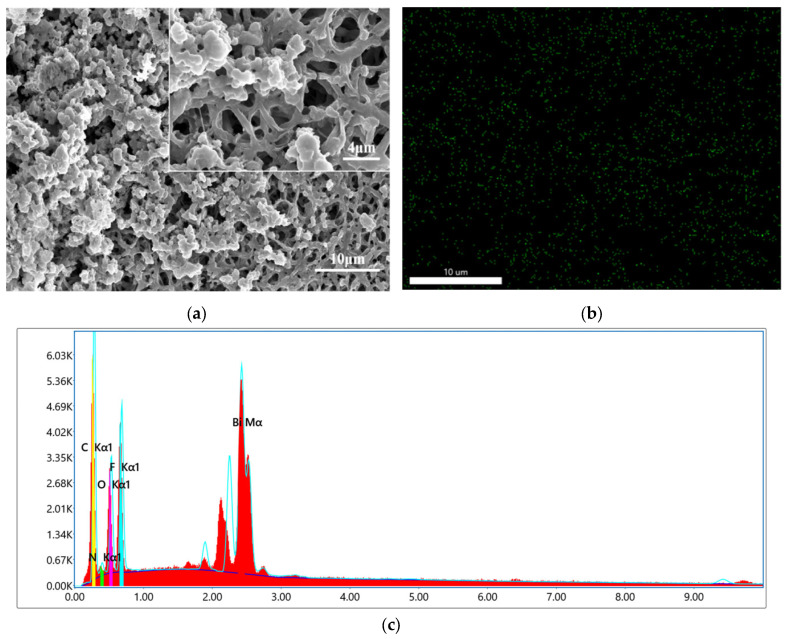

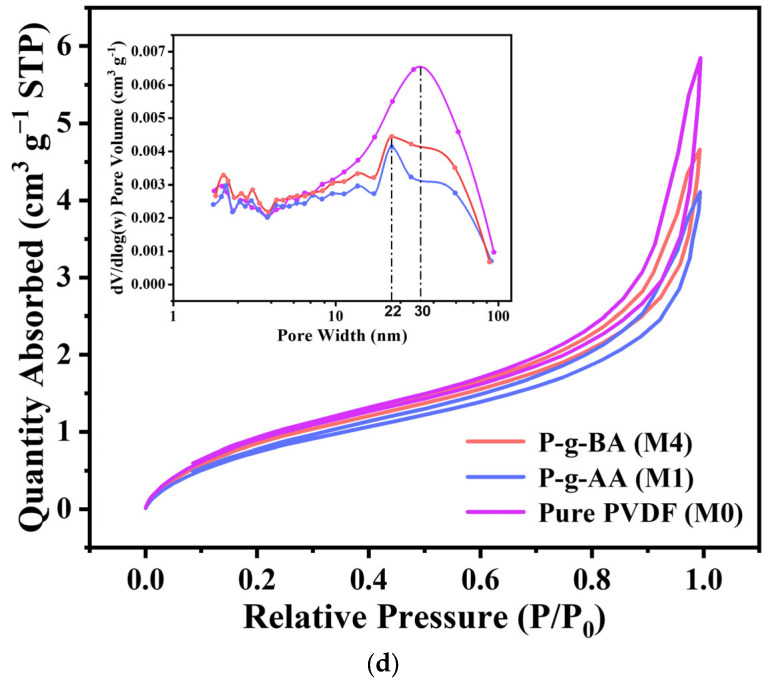
(**a**) SEM images of M4 after adsorption (the inset shows the images at a higher magnification); (**b**) the EDS map of Figure 4c for element nitrogen; (**c**) EDS pattern for the M4 after adsorption; (**d**) the changes in pore width for three types of membranes.

To explore the physical adsorption performance of the samples, we first examined the topography and atomic composition of the surfaces using SEM-EDS and then calculated their average pore size and specific surface area through adsorption/desorption experiments. The synthesized photocatalytic membrane was observed to have a planar porous loose morphology. Figure 4a shows an image of the pristine photocatalytic membrane M4 with loose and porous surfaces. Figure 4b shows the distribution of nitrogen elements on the catalytic membrane. Then, the EDS image of M4 after adsorption in Figure 4c shows the appearance of the nitrogen element compared to Figure S4, which proves that the dyes have been adsorbed onto the surface.

As shown, the adsorption/desorption isotherms are presented and determined to be type II with a H3-type hysteresis loop in Figure 4d. The calculated BET specific surface area of the samples is 3.5392, 2.9914, and 2.6824 m^2^g^−1^. It can be observed from the pore size distribution diagram that the average pore width exceeds 20 nm, specifically 30, 22, and 22 nm. This corresponds to the scanning electron microscopy analysis mentioned above. With the introduction of the catalyst, the membrane pores were blocked and the pore size decreased. However, as a dense grafted layer gradually formed on the membrane surface, the pores were reduced, leading to a decrease in roughness and water contact angle. Consequently, the hydrophilicity of M4 increased, resulting in better adsorption of water-soluble dyes. Though the small specific surface area may cause poor adsorption of dyes, a narrower pore width may also lead to the enrichment of MV and may provide sufficient active sites for the inhomogeneous reaction process.

##### Electrostatic Attraction

**Figure 5:**
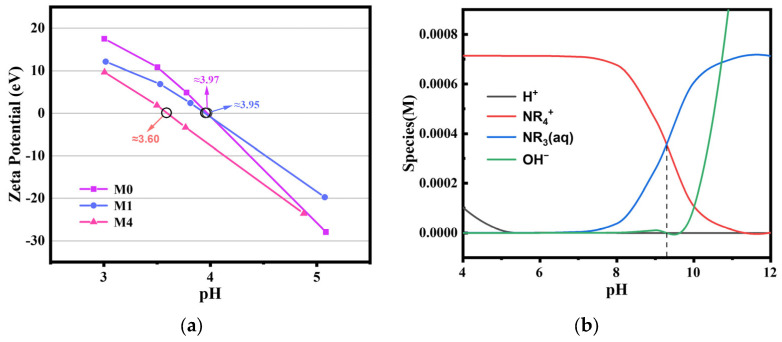
(**a**) The changes in zeta potential for the three types of membranes as pH increases; (**b**) the analog simulation of species distribution in the MV solution as pH increases.

In addition to the above physical adsorption, it is also possible that there is a weak electrostatic attraction between the membranes and the dye contaminants. From Figure 5a, it is seen that the potential value of the surface of the three types of membranes reveals their charged properties at different pH levels. It can be concluded that an increase in pH can enhance the electrostatic attraction between the MV contaminant and membrane. As can be seen from Figure 5b, when 4 < pH < 9.4, a higher NR_4_^+^ ion content in the dye solution can be attracted to the membrane surface because of the high negative charge in this range of pH, which exhibits stronger electrostatic attraction at present.

#### 3.2.2. Capacity of Chemical Adsorption

**Figure 6:**
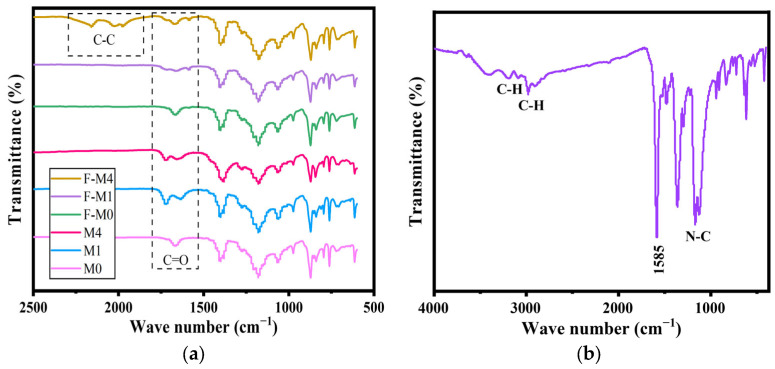
FTIR spectra of (**a**) three types of contaminated membranes, initial membranes, and (**b**) MV contaminant.

Besides the possible physical adsorption mechanisms described above, the membrane samples may also chemically bond with the adsorbed solution. Since the chemical bonding between the adsorbent and adsorbate was significantly influenced by the functional groups, Fourier infrared spectroscopy analysis of the membrane was carried out to explore the primary functional groups involved in the adsorption process. As shown in Figure 6b, we tested the infrared spectrum of the adsorbate, which shows the benzene ring C-H bond stretching vibration peak at 3191 cm^−1^, the benzene ring skeleton vibration peak at 1584 cm^−1^, and the N-C bond stretching vibration peaks at 1128 and 1168 cm^−1^.

However, in Figure 6b, the IR spectra of the modified membrane described previously are shown. We put this adsorbent in the adsorbate and obtained the infrared spectrum after adsorption, i.e., Figure 6a. When adsorbed by the adsorbent, it was found that the carbonyl stretching vibration peak appeared red-shifted from 1722 to 1672 cm^−1^, and a new set of absorption peaks appeared, with possible absorption peaks for carbon–carbon triple bonds shown in the dashed box [31]. These all indicate that some kind of chemical bonding is produced between the membrane and the adsorbate, which improves the adsorption effect.

#### 3.2.3. Dynamic Analysis

The study of dynamics is also indispensable for further elucidation of the adsorption mechanism. To study the mechanism, we calculated the dynamic data of MV adsorption to the catalyst with pseudo-first-order and pseudo-second-order simulations [32,33,34]. The formula for calculating the adsorption dynamics is shown in Supplementary Materials (S7). As a result, the calculated dynamic data (Table S2) revealed that the dynamics of the adsorption were more accurately depicted by second-order dynamics, suggesting that chemisorption constituted the rate-determining step.

Furthermore, the adsorption process can also be represented by thermodynamics. If ΔH is less than 0, the adsorption process is spontaneous and exothermic. In summary, the adsorption process was primarily influenced by Van der Waals forces, electrostatic attraction, and surface bonding, while among these three adsorption mechanisms, chemical adsorption is the rate-controlling step, which is also the main adsorption mode in the adsorption process.

### 3.3. Photocatalytic Self-Cleaning Mechanism

**Figure 7:**
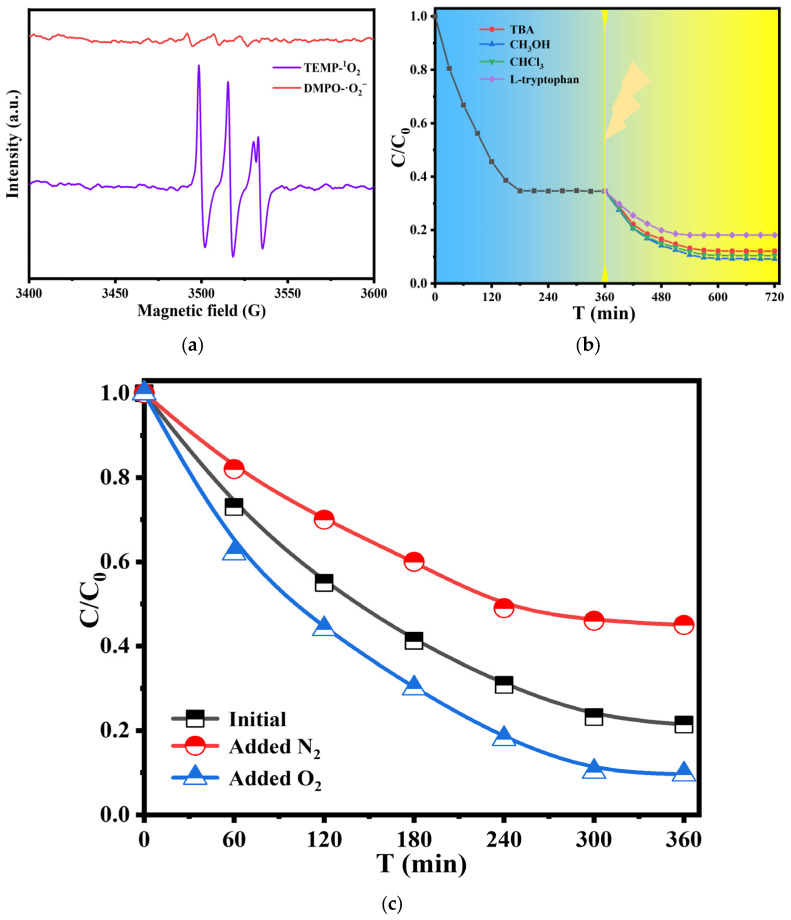
(**a**) EPR spectra of PVDF-g-BA membrane; (**b**) free radical inhibition experiments using four types of photocatalytic inhibitors; (**c**) the effect of oxygen content on photocatalytic processes.

**Scheme 1:**
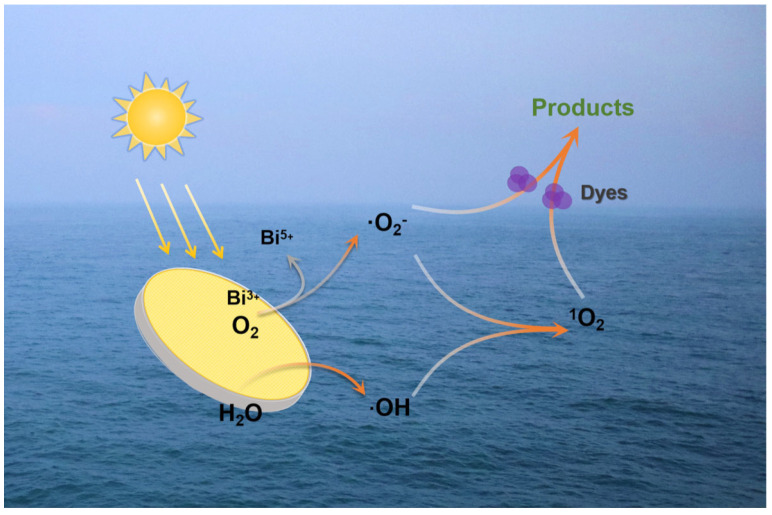
Photocatalytic degradation of MV by Bi_2_O_3_ nanoparticles.

Electron paramagnetic resonance (EPR) analysis was conducted to identify the relevant radical species using DMPO and TEMP as spin trap agents. As shown in Figure 7a, almost no signal for ·O_2_^−^ was detected. Taking into account the generation of singlet oxygen during the reaction process, we have carried out a controlled experiment with TEMP as the capture agent to establish whether the catalyst can facilitate the generation of singlet oxygen. The peak of singlet oxygen appears in the test image, indicating the presence of ^1^O_2_. The findings were in line with previous observations [35,36]. To demonstrate the validity of the above tests, a series of free radical inhibition experiments have been conducted, as shown in Figure 7b. In these inhibitors, L-tryptophan have the best inhibitory effect, TBA and CHCl_3_ show a little inhibition ability, and the CH_3_OH shows almost no ability for inhibition, which indicates that the photocatalytic process contains a large amount of ^1^O_2_ free radicals and probably has a small amount of ·OH and ·O_2_^−^ [37,38]. At last, we have found that increasing oxygen content can further enhance the photocatalytic degradation of contaminants by testing the effect of different oxygen contents on catalytic performance. Finally, we concluded that the photocatalytic mechanism of the membrane is mainly based on the oxygen element. The MV removal mechanism by PVDF-g-BA membrane could be simply explained by Scheme 1. Specifically, under sunlight irradiation, the photocatalyst Bi_2_O_3_ on the membrane absorbs solar energy and generates charge transfer in the presence of oxygen, producing superoxide radicals. Water molecules in the dye solution undergo photocatalytic degradation to generate hydroxyl radicals. These two radicals combine to form singlet oxygen. Under the action of singlet oxygen and other oxygen radicals, the dye is decomposed into renewable, clean products, with carbon dioxide being emitted. The generated water molecules can then continue to be used in the self-cleaning process, achieving a renewable self-cleaning cyclic degradation of contaminants.

Thus, a possible photocatalytic mechanism [30] involving the effect of Bi_2_O_3_ of the PVDF-g-BA membrane can be summarized by the following equations:

H_2_O → H^+^ + e^−^ + ·OH (1)

Bi^3+^ + O_2_ → Bi^5+^ + ·O_2_^−^ (2)

·O^2−^ + OH· → OH^−^ + ^1^O_2_ (3)

O_2_^−^/^1^O_2_ + dyes → Products (4)

## 4. Conclusions

In this work, we have confirmed that Bi_2_O_3_ is a visible light-responsive material, and fabricated polyvinylidene fluoride-bismuth oxide and acrylic acid (PVDF-g-BA) self-cleaning microfiltration membranes by a two-step method of adsorption deposition and ultraviolet (UV) light graft polymerization, which possesses high photocatalytic activity. Compared with the pure membrane, the PVDF-g-BA-modified membrane with 0.5% Bi_2_O_3_ not only enhances the filtering performance, but also has strong anti-fouling performance, recycling capacity, and self-cleaning ability. Furthermore, the degradation effect of the organic dye methyl violet 2B (one of crystal violet dyes) under visible light irradiation has turned out to be very significant. The adsorption and photocatalytic degradation rate can reach 90%, and after 12 h, the dyes were almost completely degraded. This modification gives the membrane a self-cleaning ability under visible light, making it potentially very valuable for water treatment.

## Figures and Tables

**Table 1:** Grafting data of modified membranes.

Membrane	Bi_2_O_3_ (mg)	AA (mg)	Bi Grafting (%)	AA Grafting (%)
M1	0	21.40	0	5.02
M4	8.86	44.94	3.57	10.15

## Data Availability

Data is contained within the article or Supplementary Materials.

## Supplementary Materials

The following supporting information can be downloaded at: https://www.mdpi.com/article/10.3390/polym16162322/s1, Figure S1. Preparation process and surface photo of PVDF-g-BA (M4). Figure S2. SEM images of M0, M1 before and after adsorption (the inset shows the images at a higher magnification). Figure S3. Surface photos of (a) M2, (b) M3, (c) M4, (d) M5 and (e) M6; SEM images of (f) M2, (g) M3, (h) M5 and (i) M6. Figure S4. EDS mapping of M4 before adsorption. Figure S5. Water flux of seven types of membranes by ten times consecutive filtration. Figure S6. SEM images of M4 after photocatalytic degradation (a) once, (b) three times and (c) five times. Table S1. Compositions and Nomenclature of Modified Membranes. Table S2. Rejection ratios of MV, MB, RhB and AO7 dyes through different modified membranes. Table S3. Comparison between the adsorption rate constants, Qe, estimated andcorrelation coefficient associated with pseudo-first-order and the pseudo-second-order rate equations.
