# Supplementary material for: Ultraviolet Grafting of Bismuth Oxide Enhances the Photocatalytic Performance of PVDF Membrane and Improves the Problem of Membrane Fouling
Source: Polymers (Basel). 2024 Aug 16;16(16):2322. doi: 10.3390/polym16162322 (PMC11359878; doi:10.3390/polym16162322)
Supplement: Supplementary file 1 [file polymers-16-02322-s001.zip › polymers-3124572-supplementary.pdf]

Supplementary Information

Ultraviolet Grafting of Bismuth Oxide Enhances the Photocatalytic Performance of PVDF Membrane and Improves the Problem of Membrane Fouling

Chang Liu [1], Yuxuan Kong [1], Guojiang Xia [1], Xiancheng Ren [1,*] and Jing Zhang [2,*]

[1] College of Polymer Science and Engineering, Sichuan University, Chengdu 610065, China
[2] College of Architecture and Environment, Sichuan University, Chengdu 610065, China
* Correspondence: xiancren@sina.com (X.R.); zjing428@163.com (J.Z.)

S1 The rate of grafting

Generally, we could obtain the equation for the total grafting rate of the membrane in many literatures. The commonly used calculation formula is shown below：

$$wt\% = (W_2 - W_1)/W_1 \times 100\% \tag{1}$$

where W_1 and W_2 are the weight of a membrane before and after grafting reaction, respectively. The rate of membrane interface grafting calculation formula in this study is derived from the literature. Because Bi_2O_3 nanoparticles were grafted on the membrane in our research, the mass of Bi_2O_3 (data obtained from Atomic Absorption Spectrometry) needs to be subtracted from the weight gain of the membrane. The final results of grafting data are shown in Table 1.

S2 *Schematic diagram of synthesis of PVDF-g-BA*

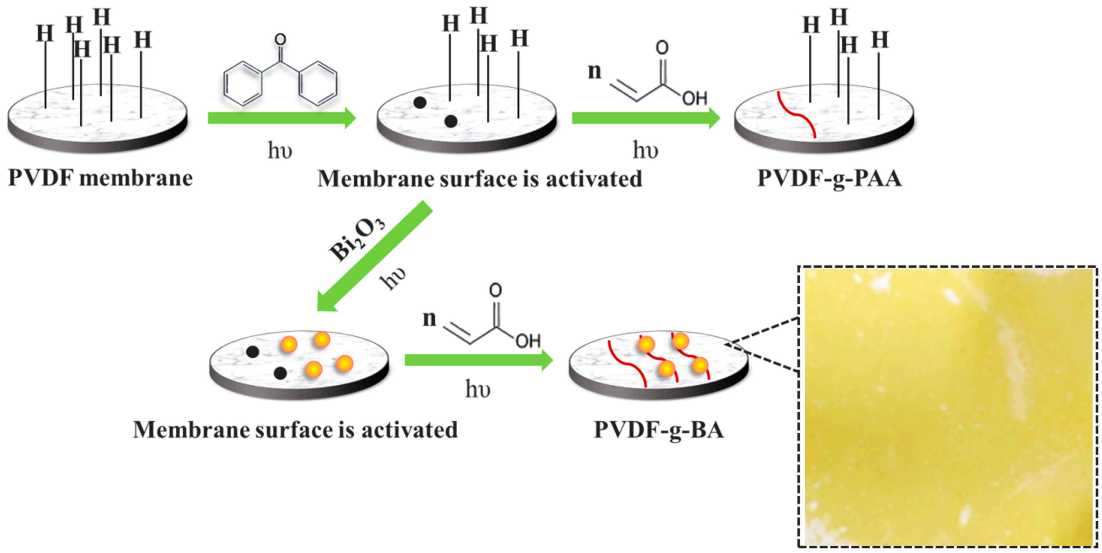

Figure S1. Preparation process and surface photo of PVDF-g-BA (M4).

S3 SEM images of M0 and M1

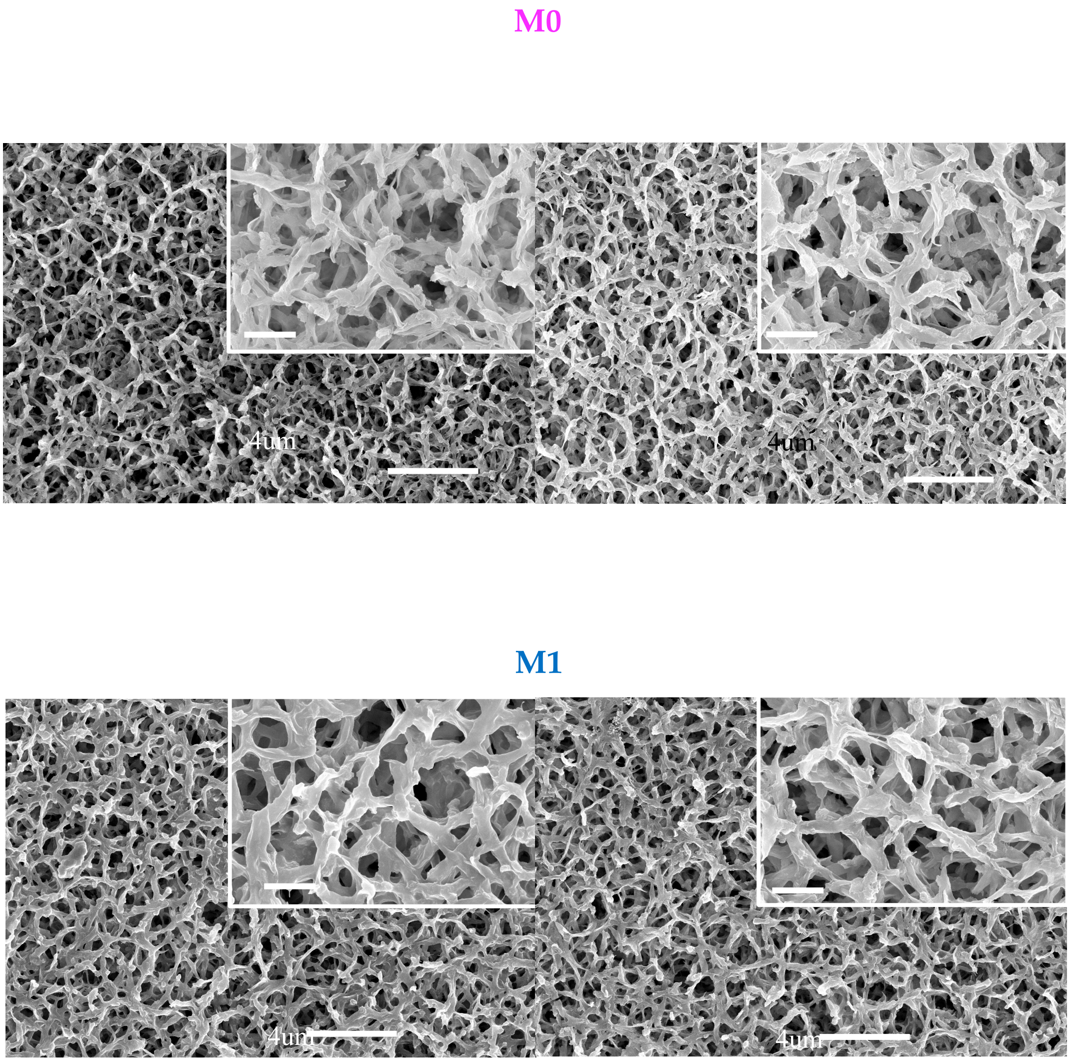

Figure S2. SEM images of M0, M1 before and after adsorption (the inset shows the images at a higher magnification).

S4 Surface morphology of M2-M6 and EDS element distribution spectrum of M4 before adsorption

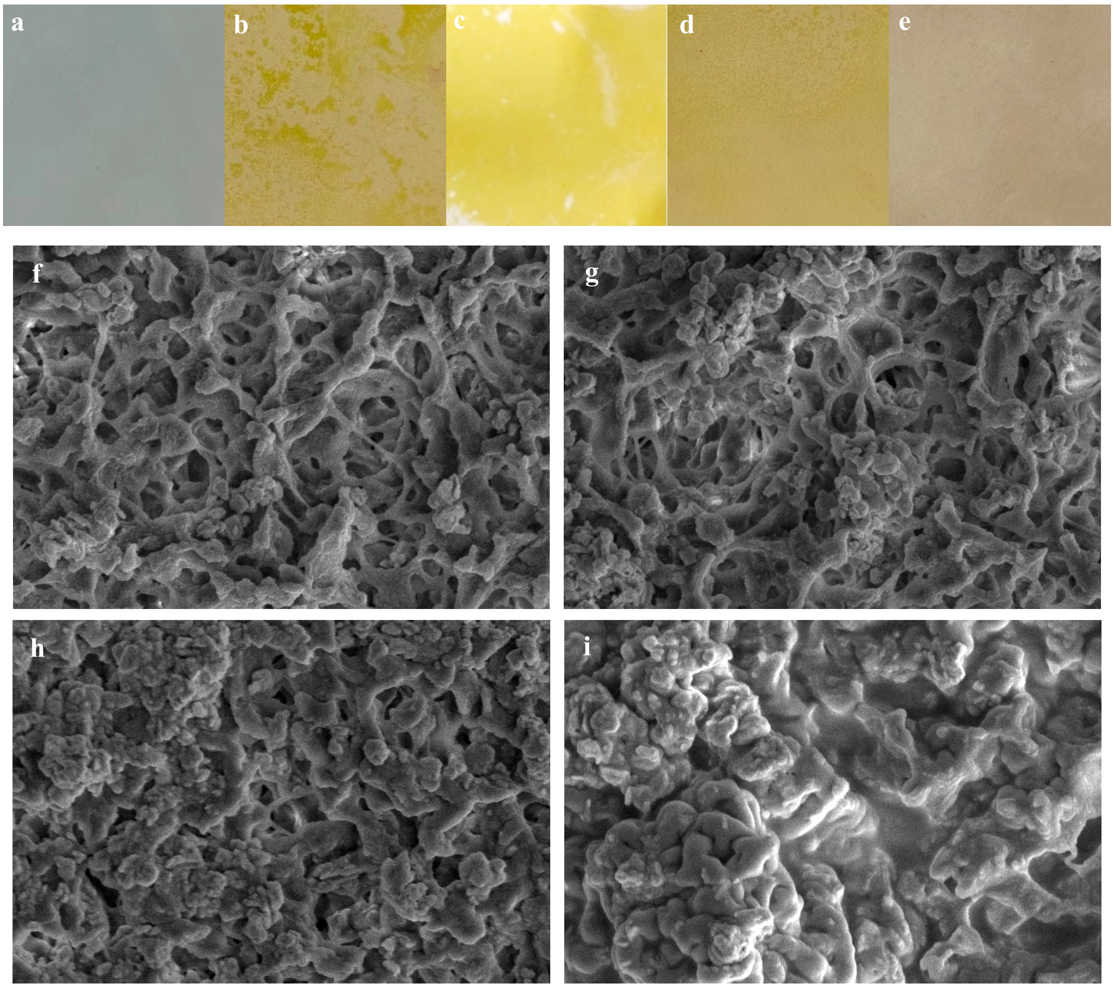

Figure S3. Surface photos of (a) M2, (b) M3, (c) M4, (d) M5 and (e) M6; SEM images of (f) M2, (g) M3, (h) M5 and (i) M6.

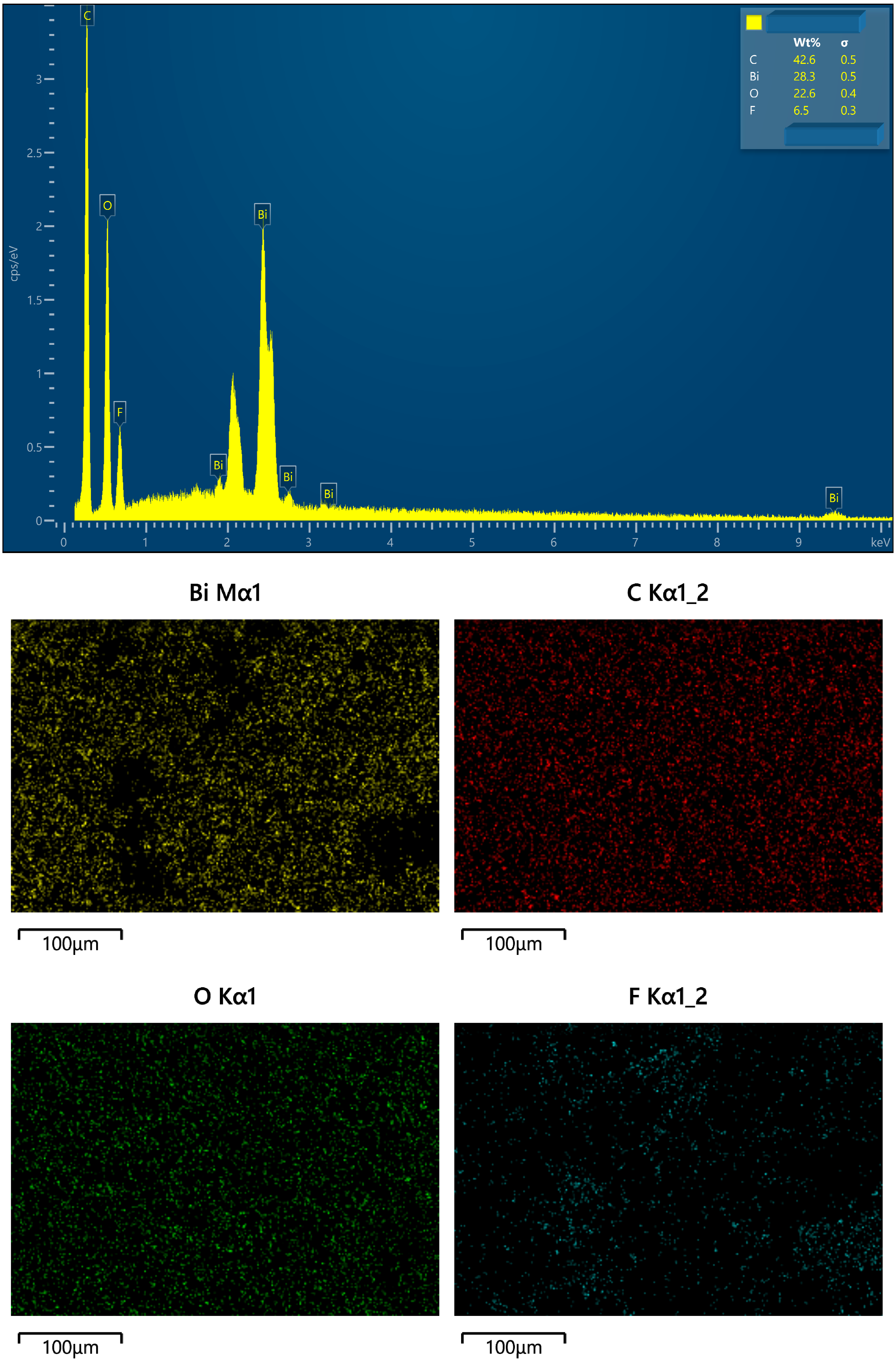

Figure S4. EDS mapping of M4 before adsorption.

S5 Water flux of seven types of membranes

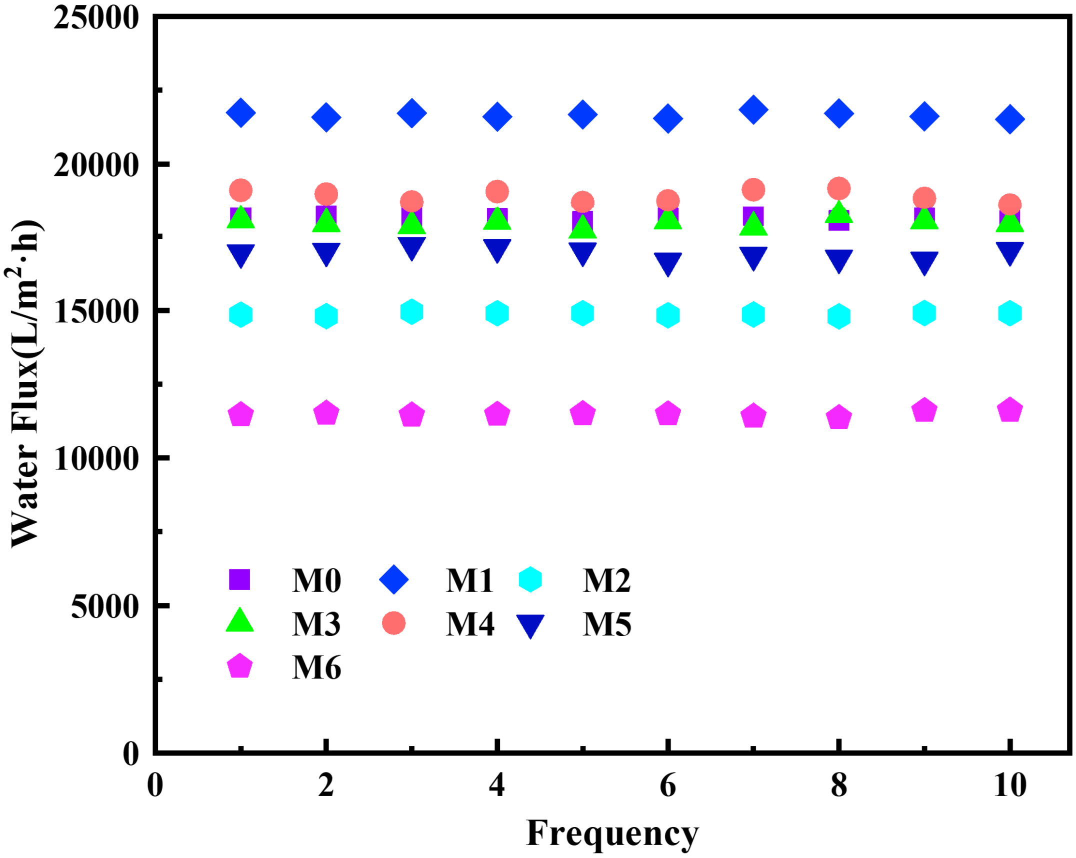

Figure S5. Water flux of seven types of membranes by ten times consecutive filtration.

S6 SEM of M4 after photocatalytic degradation once, three times and five times

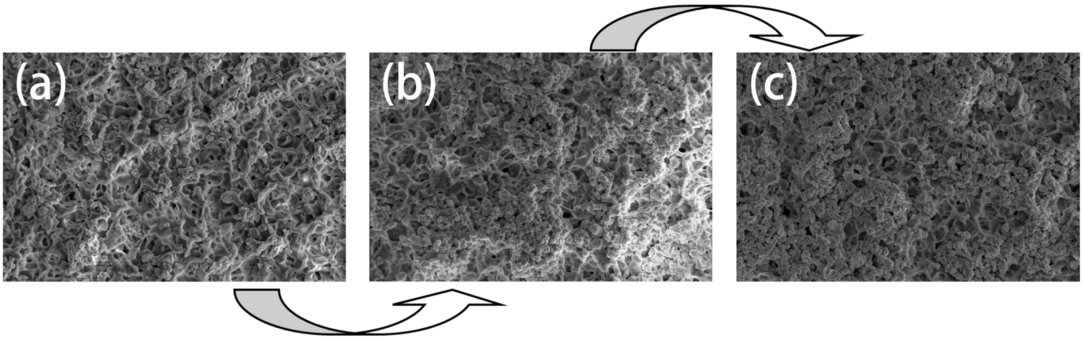

Figure S6. SEM images of M4 after photocatalytic degradation (a) once, (b) three times and (c) five times.

S7 Adsorption kinetics simulation method

The quantity of MV absorbed onto the catalysts at time t [Q_t (mg/g)] was calculated by (2). The MV adsorption amount at equilibrium, Q_e (mg/g), was calculated by (3), where C_0 and C_t and C_e (all in the unit of mg/L) are MV concentrations initially, at time t, and equilibrium. And V (L) and W (g) are the MV solution volume and the adsorbent mass, respectively. Where k_1 (min^{-1}) and k_2 (g·mg^{-1}·min^{-1}) are the adsorption rate constants of the pseudo-first-order and the pseudo-second-order models, respectively. The final results of adsorption kinetics are shown in Table S2.

$$Q_t = (C_0 - C_t) \times V / W \quad (2)$$

$$Q_e = (C_0 - C_e) \times V / \quad (3)$$

$$log(Q_e - Q_t) = logQ_e - k_1 \times t / 2.303 \quad (4)$$

$$t / Q_t = 1 / (k_2 \times Q_e^2) + t / \quad (5)$$

Table S1 Compositions and Nomenclature of Modified Membranes

membrane	Bi_2O_3 (wt%)	AA (wt%)
M0	0	0
M1	0	20
M2	0.10	20
M3	0.25	20
M4	0.50	20
M5	0.75	20
M6	0.90	20

Table S2 Rejection ratios of MV, MB, RhB and AO7 dyes through different modified membranes

Dyes\Membranes	M0	M1	M4
**Rejection(MV 584nm)/%**	38.2	56.5	95.4
**Rejection(MB 664nm)/%**	60.8	75.4	90.3
**Rejection(RhB 552nm)/%**	40.6	55.2	82.5
**Rejection(AO7 484nm)/%**	45.4	59.8	77.6

Table S3 Comparison between the adsorption rate constants, Q_e, estimated andcorrelation coefficient associated with pseudo-first-order and the pseudo-second-order rate equations.

Initial dye concentration (mg/L)	k_1 (min^{-1})	Q_e (mg/g)	R^2	k_2 (g $mg^{-1}min^{-1}$)	Q_e (mg/g)	R^2
M4(3.8)	0.209	2.125	0.9925	0.0089	25.362	0.9939
M1(3.8)	0.288	1.484	0.9896	0.0076	21.125	0.9965
M0(3.8)	0.562	0.895	0.9912	0.0054	10.526	0.9897
